# Vitamin D3 regulates PM-driven primary human neutrophil inflammatory responses

**DOI:** 10.1038/s41598-023-43252-1

**Published:** 2023-09-22

**Authors:** Chidchamai Kewcharoenwong, Aranya Khongmee, Arnone Nithichanon, Tanapat Palaga, Tassanee Prueksasit, Ian S. Mudway, Catherine M. Hawrylowicz, Ganjana Lertmemongkolchai

**Affiliations:** 1 Department of Medical Technology, Faculty of Associated Medical Sciences, Chiang Mai University, Chiang Mai, 50200 Thailand; 2 Centre for Research and Development of Medical Diagnostic Laboratories, Faculty of Associated Medical Sciences, Khon Kaen University, Khon Kaen, 40002 Thailand; 3 Department of Microbiology, Faculty of Medicine, Khon Kaen University, Khon Kaen, 40002 Thailand; 4 Department of Microbiology, Faculty of Science, Chulalongkorn University, Bangkok, 10330 Thailand; 5 Department of Environmental Science, Faculty of Science, Chulalongkorn University, Bangkok, 10330 Thailand; 6 MRC Centre for Environment and Health, School of Public Health, Imperial College London, London, UK; 7 King’s Centre for Lung Health, School of Immunology and Microbial Sciences, King’s College London, London, W2 1PG UK; 8 National Institute of Health Research, Health Protection Research Unit in Environmental Exposures and Health, Imperial College London and King’s College London, London, W12 OBZ UK

**Keywords:** Immunology, Microbiology, Environmental sciences, Medical research

## Abstract

Recent evidence has demonstrated that both acute and chronic exposure to particulate air pollution are risk factors for respiratory tract infections and increased mortality from sepsis. There is therefore an urgent need to establish the impact of ambient particulate matter (PM) on innate immune cells and to establish potential strategies to mitigate against adverse effects. PM has previously been reported to have potential adverse effects on neutrophil function. In the present study, we investigated the impact of standard urban PM (SRM1648a, NIST) and PM_2.5_ collected from Chiang Mai, Thailand, on human peripheral blood neutrophil functions, including LPS-induced migration, IL-8 production, and bacterial killing. Both NIST and the PM_2.5_, being collected in Chiang Mai, Thailand, increased IL-8 production, but reduced CXCR2 expression and migration of human primary neutrophils stimulated with *Escherichia coli* LPS. Moreover, PM-pretreated neutrophils from vitamin D-insufficient participants showed reduced *E. coli*-killing activity. Furthermore, in vitro vitamin D3 supplementation attenuated IL-8 production and improved bacterial killing by cells from vitamin D-insufficient participants. Our findings suggest that provision of vitamin D to individuals with insufficiency may attenuate adverse acute neutrophilic responses to ambient PM.

## Introduction

Long-term exposure to PM, especially the fine fraction (PM_2.5_), is associated with increased risk of cardiovascular disease, diabetes, acute respiratory tract infections (RTIs)^1–3^ and sepsis-related mortality^4^. All these conditions are linked to local airway and systemic inflammation, which are induced following both short^5,6^ and long^7–9^ term air pollution exposures. Both PM_10_ and PM_2.5_ have been reported to have adverse effects on airway structural and immune cells, including both the recruitment and activation of neutrophils at the epithelial barriers encompassing respiratory tract lining fluid and resident inflammatory cells^10,11^. After exposure to PM_2.5_, neutrophils are recruited into the lungs of mice and associated with production of proinflammatory cytokines^12^. IL-8 or CXCL8 is the most potent neutrophil-recruiting chemokine^13^. Human exposures to freshly generated diesel exhaust have also reported IL-8 production in the airways, concomitant with increased neutrophil infiltration into the bronchial mucosa and central airways and sustained up to 18 h post exposure^14–16^.

Moreover, cigarette smoke PM challenge has been shown to compromise *Escherichia coli* LPS-induced proinflammatory cytokine production and the bacterial killing function of murine neutrophils^17^. During infection, Toll-like receptor 4 (TLR4) responds to the presence of LPS at the site of infection or bloodstream and triggers pro-inflammatory reactions, for instance cytokine/chemokine production, facilitating elimination of the invading gram-negative bacteria^18^. Nevertheless, relatively few studies have focused on primary human neutrophil responses following particulate pollutant challenge, and the underlying mechanisms by which inhaled PM may affect neutrophil function in response to infection remain unclear.

Vitamin D is a secosteroid hormone that is well known for its role in mineral and skeletal homeostasis. Vitamin D also plays a critical role in modulating the immune response, including the response to respiratory infections^19^. Vitamin D deficiency has been associated with disease severity in community-acquired pneumonia^20^ and other RTIs^21^, while vitamin D supplementation has proven benefits in patients with these conditions^22^. Moreover, vitamin D is an essential component of innate immune modulation^19,23^: it promotes pneumococcal killing and modulates inflammatory responses in primary human neutrophils^24^. Furthermore, vitamin D3 may reduce the predominantly oxidative stress-mediated inflammation induced by PM^25,26^, and vitamin D3 has been shown to increase human α-1-antitrypsin (AAT), a serine protease inhibitor with immunoregulatory properties that prevents excessive lung damage by neutrophil elastase, in CD4 + T cells^27,28^. Based on these previous observations we hypothesized that vitamin D3 would modulate the inflammatory responses of PM-exposed neutrophils. We therefore examined whether vitamin D3 was protective against a range of PM-driven human neutrophil responses relevant to infection risk: the production of IL-8, cell migration and bacterial killing.

## Results

### PM enhances interleukin-8 production by primary human neutrophils in response to lipopolysaccharide (LPS) challenge

**Figure 1:**
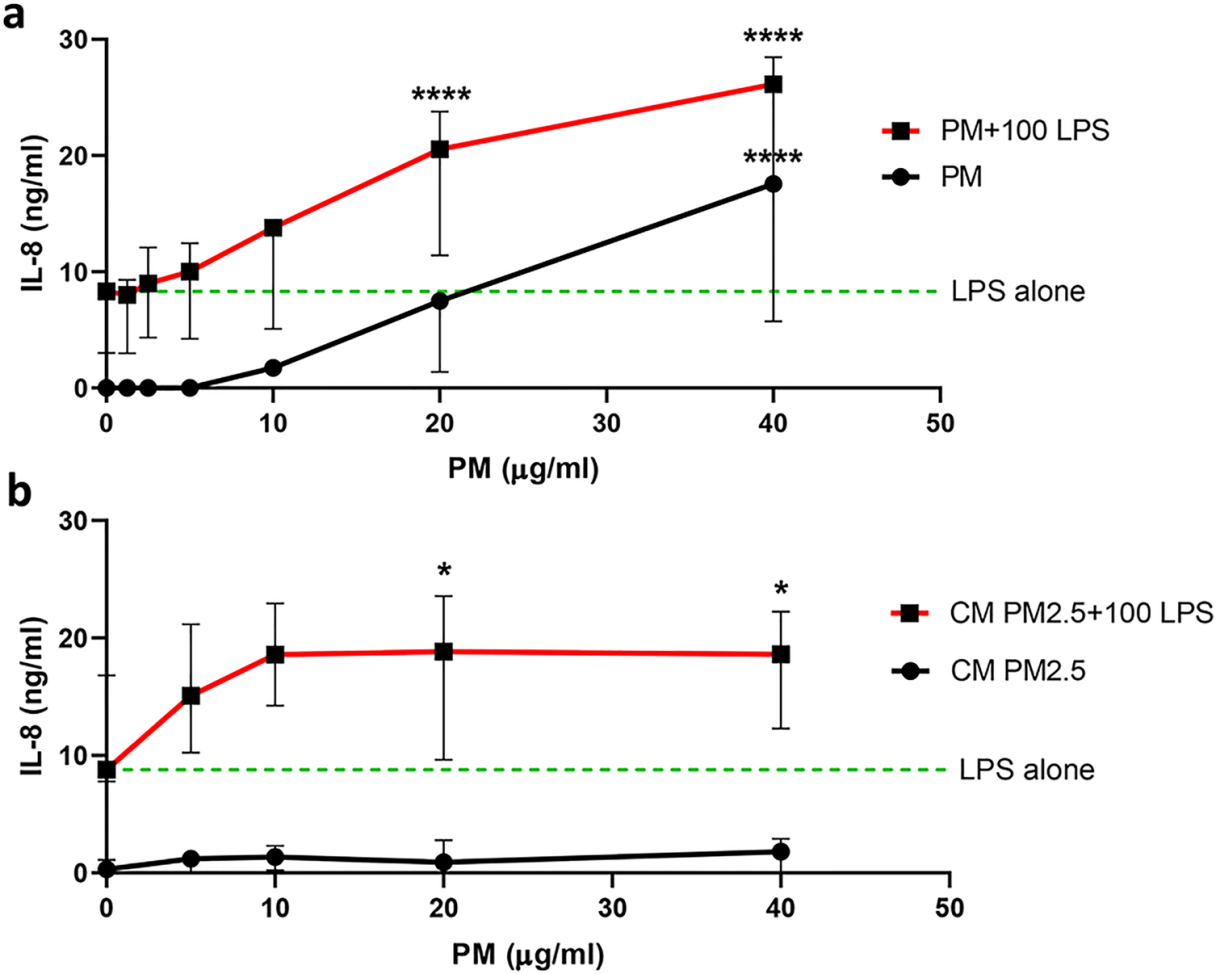
PM enhances IL-8 production by human neutrophils in response to LPS. Isolated human neutrophils at 2.5 × 10^6^ cells/ml from vitamin D-sufficient participants (n = 3) were pretreated with (**a**) SRM 1648a (PM; 0, 5, 10, 20 and 40 μg/ml) or (**b**) Chiang Mai PM_2.5_ (CM PM_2.5_; 0, 1.25, 2.5, 5, 10, 20 and 40 μg/ml) with or without 100 ng/ml *E. coli* LPS stimulation. The supernatant of PM-pretreated neutrophil cultures was harvested at 18 h for IL-8 detection. Each dot indicates the median with the range. Asterisks indicate significant differences between PM with LPS condition (red line) versus LPS alone stimulation (green dashed line) or between PM alone (black line) and medium control at 0 μg/ml PM. Statistical analysis was performed using two-way ANOVA with Šídák’s multiple comparisons test. ****P < 0.001, No asterisk- nonsignificant.

Isolated neutrophils from healthy participants were pre-treated with standard reference urban PM (SRM 1648a) prior to stimulation with LPS for 18 h. Initial experiments confirmed no loss of cell viability with PM incubation at concentrations ranging from 1.25 to 40 µg/ml for 30, 60 and 90 min (Supplementary Table S1). We observed that standard urban PM alone significantly induced IL-8 production in a dose-dependent manner (Fig. 1a). Moreover, pretreatment of neutrophils with standard urban PM led to an additive effect of IL-8 production in response to LPS compared with LPS stimulation alone, which was statistically significant at 20 and 40 µg/ml (Fig. 1a). We further determined these effects with Chiang Mai PM_2.5_. Although treatment with Chiang Mai PM_2.5_ alone did not induce a significant increase in IL-8 production over the range of concentrations tested, enhancement of IL-8 production in response to LPS compared with LPS stimulation alone was similar to that induced by standard urban PM (Fig. 1b). There was insufficient quantity of the CM PM_2.5_ sample for further comprehensive analyses and therefore all further experiments were conducted using SRM 1648a.

### Pretreatment of primary human neutrophils with PM decreased expression of the IL-8 receptor and reduced the migration index in response to LPS

**Figure 2:**
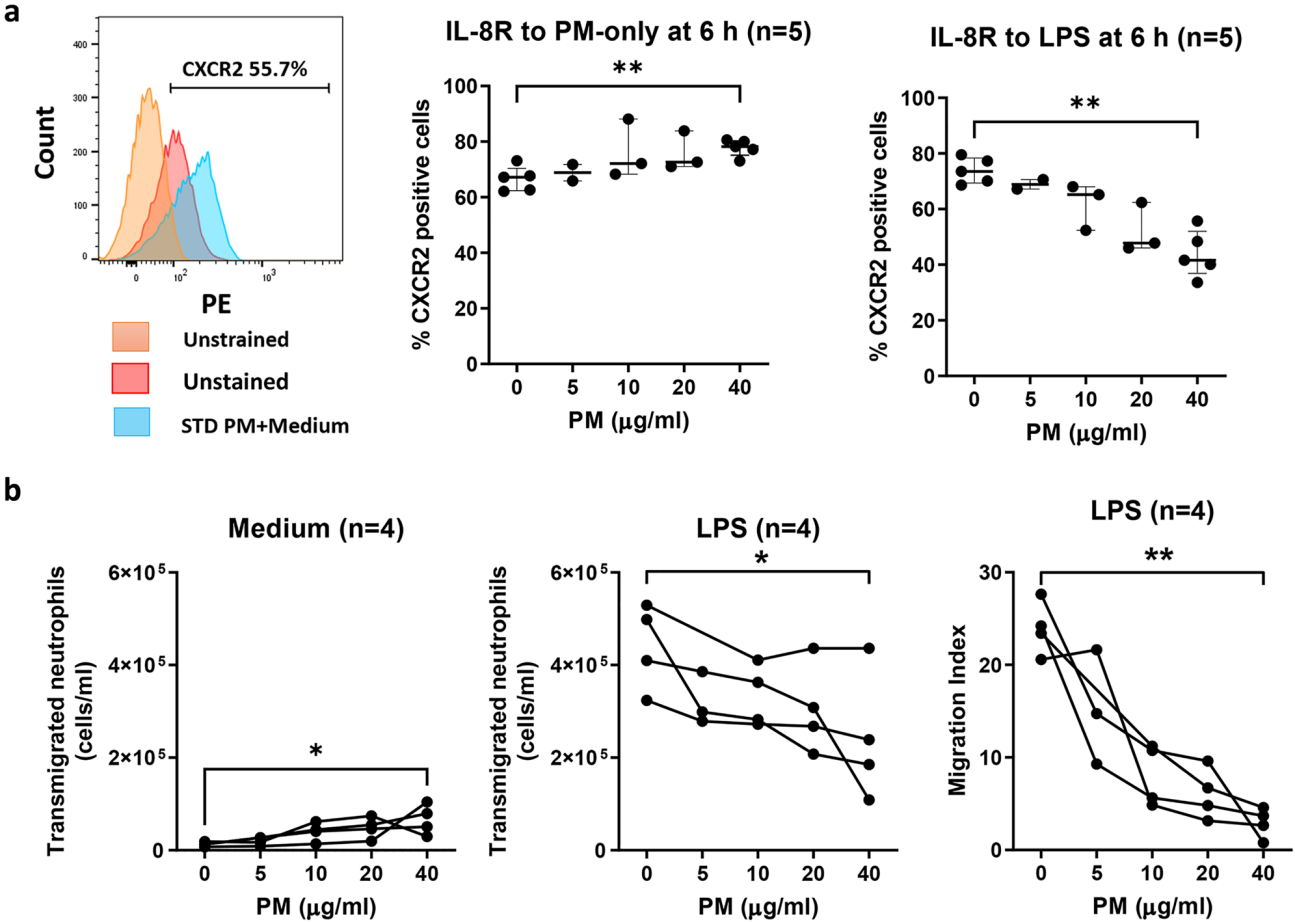
Standard urban PM reduces LPS-induced CXCR2 expression and migration of human neutrophils in response to *E. coli* LPS. Isolated human neutrophils at 2.5 × 10^6^ cells/ml from vitamin D-sufficient participants were pretreated with SRM 1648a (PM; 0, 5, 10, 20 and 40 μg/ml) with or without 100 ng/ml *E. coli* LPS stimulation. (**a**) After 6 h, the cells (n = 5) were stained with anti-human CXCR2-PE and analysed by flow cytometry by gating the percentage of CXCR2-expressing cells compared with unstained cells from the selected neutrophil population. Percentage enhancement was calculated by comparison with the no PM condition. Each bar represents the median with interquartile range. (**b**) SRM 1648a—pretreated neutrophils (n = 4) were added to upper wells and cocultured with *E. coli* LPS in lower wells. After coculture for 1 h, transmigrating neutrophils were counted by flow cytometry. The dark circles indicate the migration index or number of transmigrated cells for neutrophils from each donor under each exposure condition. Statistical analysis was performed using Friedman’s test with Dunn’s post hoc analysis to compare among PM concentrations, **P < 0.01.

We further investigated the effect of standard (SRM 1648a) PM on downstream functions affected by IL-8 production, specifically IL-8 receptor (CXCR2) expression and neutrophil migration activity. Isolated neutrophils were pretreated with PM at a range of concentrations, with or without 100 ng/ml LPS stimulation. After 6 h, the cells were stained with anti-human CXCR2-PE and analysed by flow cytometry. We found little evidence that PM alone enhanced the frequency of CXCR2-expressing neutrophils over the concentration range 5–40 ug/ml, though a statistically significant increase was evidence at the highest (40 ug/ml) dose. However, PM pretreatment with LPS stimulation significantly reduced LPS-induced CXCR2 expression in primary human neutrophils in a dose-dependent manner (Fig. 2a).

To determine neutrophil migration activity by Transwell plate, standard (SRM 1648a) PM-pretreated neutrophils were added to the upper wells and cocultured with LPS or medium alone as the control, which was added to the lower wells. The data are represented as both the number of transmigrated neutrophils and the migration index, which was calculated by subtracting the absolute count of transmigrated neutrophils to LPS from the medium control and then dividing this by transmigrated neutrophils in the medium control (Fig. 2b). Firstly, in PM-pretreated neutrophils cocultured with medium alone, the number of transmigrated neutrophils was modestly, but significantly increased at the highest PM concentration tested (Fig. 2b; 1st panel). In contrast, LPS-induced neutrophil migration was much stronger than that induced by PM, and LPS-induced neutrophil migration was inhibited by PM pretreatment in a dose-dependent manner (Fig. 2b). These data indicate that PM treatment alone activates neutrophil functions, but impairs the migration activity of primary human neutrophils in response to LPS.

### 1α,25-Dihydroxyvitamin D3 reduces the inflammatory responses of primary human neutrophils pre-treated with PM samples

**Figure 3:**
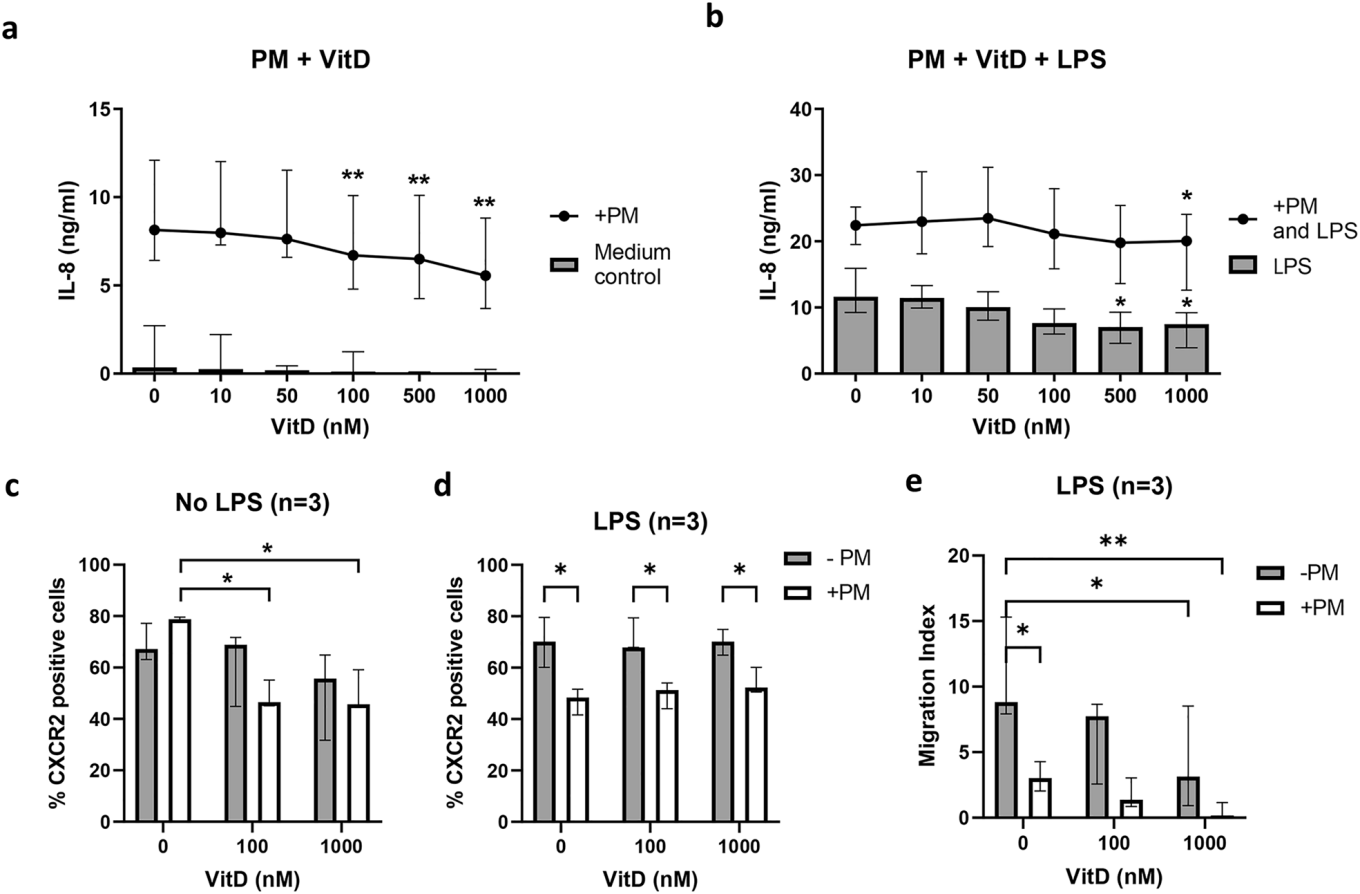
1α,25-Dihydroxyvitamin D3 reduces the inflammatory response of PM-treated human neutrophils. Isolated human neutrophils at 2.5 × 10^6^ cells/ml from vitamin D-sufficient participants (n = 3) were pretreated with 40 μg/ml SRM 1648a and varying concentrations of 1α,25-dihydroxyvitamin D3 (Vit D; 0, 10, 50, 100, 500 and 1000 nM) for 30 min and then restimulated with (panel (**a**)) medium control (no LPS) or (panel (**b**)) 100 ng/ml *E. coli* LPS. The supernatant of pretreated neutrophil cultures was harvested at 18 h for IL-8 detection. Each dot indicates the median with the range of IL-8 concentrations. Statistical analysis was performed using two-way ANOVA with Šídák's multiple comparisons test between the medium control and 1α,25-dihydroxyvitamin D3 treatments. A cell suspension of pretreated neutrophil cultures at 6 h was stained with anti-human CXCR2-PE and analysed by flow cytometry (panels (**c, d**)). Each bar indicates the median with the range of % CXCR2-positive cells. The pretreated cells were added to upper wells and cocultured with *E. coli* LPS in lower wells. After coculture for 1 h, transmigrating neutrophils were counted by flow cytometry, and the migration index was calculated (panel e). Each bar indicates the median with the range of the neutrophil migration index. Grey and white bars indicate untreated and PM-treated conditions, respectively. Statistical analysis was performed using two-way ANOVA with Šídák's multiple comparisons test. **P < 0.01, *P < 0.05.

To determine whether 1α,25-dihydroxyvitamin D3 modulates IL-8 production, neutrophils were pretreated with 40 μg/ml PM (SRM 1648a) and various concentrations of 1α,25-dihydroxyvitamin D3. Initial experiments were performed using neutrophils isolated from vitamin D-sufficient participants (Table 1). Production of IL-8 induced by PM was significantly reduced in neutrophils by 1α,25-dihydroxyvitamin D3 in cell culture (Fig. 3a). Next, we investigated the effect of vitamin D3 on PM-pretreated neutrophils in response to LPS, and a similar reduction was observed with LPS-induced IL-8 production, as represented by bar graphs in Fig. 3b. Moreover, in neutrophils pretreated with both PM and the highest concentration of 1α,25-dihydroxyvitamin D3 studied, IL-8 production was modestly, but significantly reduced in response to LPS exposure, as represented by line-dot plots in Fig. 3b.

**Table 1:** Characteristics of the human participants.

Participants	N	Gender (F:M)	Age (year ± SD)	Vitamin D level (free 25-OH, pg/ml, mean ± SD)
Vit D sufficient	10	5:5	52 ± 12	9.6 ± 1.6
Vit D insufficient	10	7:3	45 ± 10	4.4 ± 0.9

Next, we observed the effect of 1α,25-dihydroxyvitamin D3 on CXCR2 expression. Without LPS addition, CXCR2 expression was significantly decreased in neutrophils pretreated with PM and 1α,25-dihydroxyvitamin D3 compared to PM alone (Fig. 3c). On the other hand, LPS exposure abrogated the effect of 1α,25-dihydroxyvitamin D3 across all conditions (Fig. 3d), and the migration index of neutrophils pretreated with 1α,25-dihydroxyvitamin D3 in response to LPS exposure was significantly reduced both with (white bars) and without (grey bars) PM pretreatment (Fig. 3e). Taken together, our data indicate that PM alone is not only proinflammatory, but also compromises the inflammatory response to TLR ligation by LPS.

### The responses of neutrophils from vitamin D-insufficient participants in response to PM challenge are attenuated by 1α,25-dihydroxyvitamin D3 treatment

We next investigated whether there were any differences in PM-induced responses in neutrophils obtained from vitamin D-sufficient and vitamin D-insufficient (< 5.8 pg/ml)^29^ participants, and the effect of 1α,25-dihydroxyvitamin D3 on this response. The free 25-OH vitamin D concentrations in plasma, which are strongly correlated to total 25-OH vitamin D in most populations^30^, were 9.6 ± 1.6 and 4.4 ± 0.9 pg/ml for vitamin D-sufficient and -insufficient subjects, respectively (Table 1).

**Figure 4:**
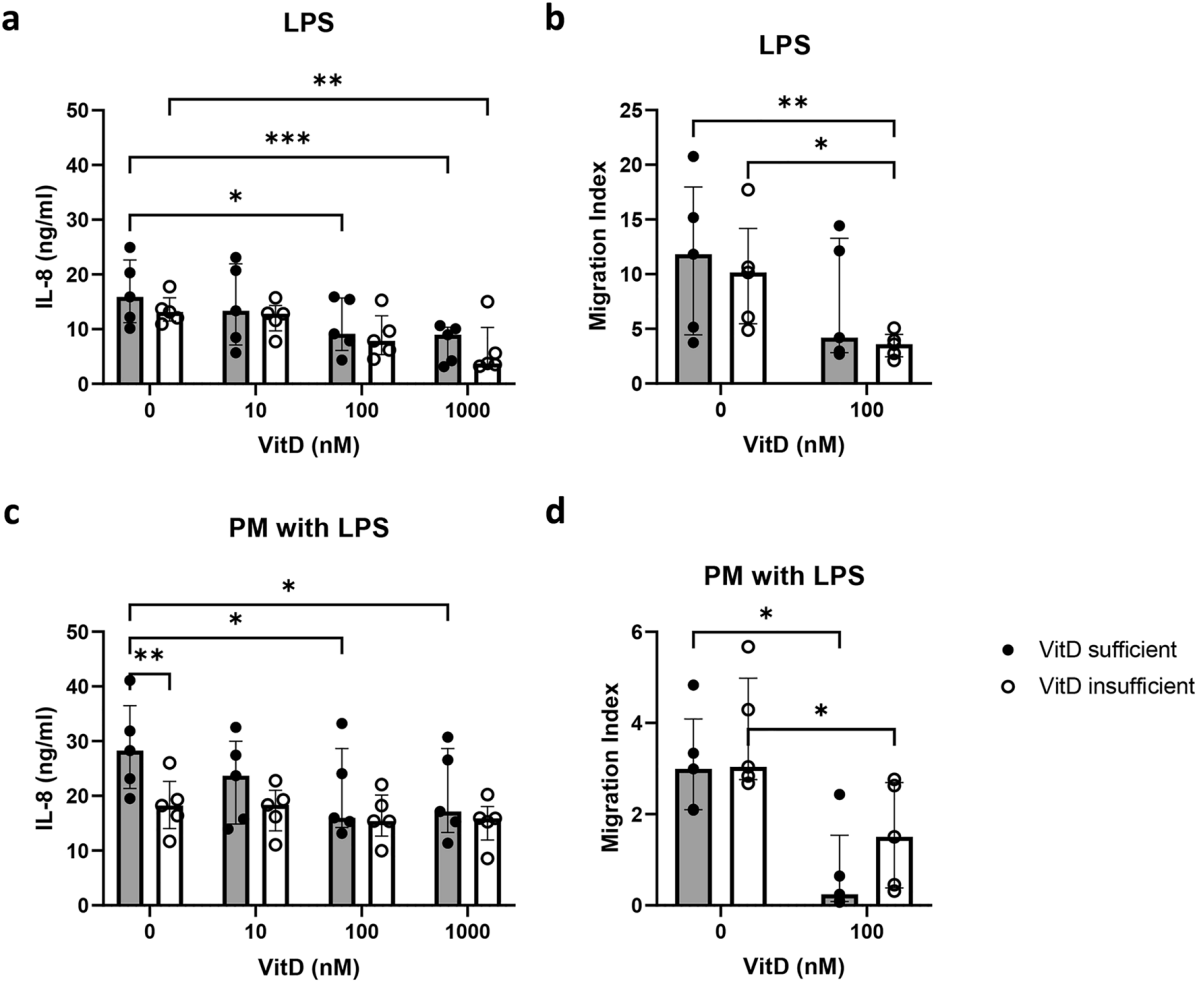
1α,25-Dihydroxyvitamin D3 reduces IL-8 production and migration of PM-treated neutrophils isolated from vitamin D-sufficient and vitamin D-insufficient participants. Isolated neutrophils at 2.5 × 10^6^ cells/ml from vitamin D-sufficient (n = 5) and vitamin D-insufficient (n = 5) participants were pretreated with 40 μg/ml standard urban PM with or without 1α,25-dihydroxyvitamin D3 (VitD; 10, 100 and 1000 nM) for 30 min and then stimulated with 100 ng/ml *E. coli* LPS. Panels (**a**) and (**c**) show median (interquartile range) IL-8 concentrations in supernatant from pretreated neutrophil cultures at 18 h. Isolated neutrophils were pretreated with 40 μg/ml standard urban PM with or without 100 nM 1α,25-dihydroxyvitamin D3 for 30 min. The pretreated cells were added to upper wells and cocultured with *E. coli* LPS in lower wells. After coculture for 1 h, transmigrating neutrophils were counted by flow cytometry. Panels (**b**) and (**d**) show the median (IQR) of the neutrophil migration index, and each dot represents the value of each participant. Grey and white bars indicate vitamin D-sufficient and vitamin D-insufficient participants, respectively. Statistical analysis was performed using two-way ANOVA with Šídák’s multiple comparisons test among 1α,25-dihydroxyvitamin D3 pretreatments and between sufficient and insufficient participants. ****P < 0.001, **P < 0.01, *P < 0.05, No asterisk- nonsignificant.

1α,25-dihydroxyvitamin D3-pretreated neutrophils showed a significantly reduced IL-8 production in response to LPS in both vitamin D-sufficient and vitamin D-insufficient participants (Fig. 4a) and a decreased migration index (Fig. 4b) that achieved significance in the vitamin D-insufficient participants only. However overall, there was no significant difference between the groups. In PM-pretreated neutrophils treated with LPS, neutrophils from vitamin D-insufficient participants secreted lower concentrations of IL-8 in response to LPS than those from vitamin D-sufficient participants. Furthermore, decreased IL-8 production due to vitamin D3 treatment was observed only in vitamin D-sufficient participants (Fig. 4c), and reduction in IL-8 production by 1α,25-dihydroxyvitamin D3 in culture in vitamin D-insufficient participants was less than that in vitamin D-sufficient participants (Supplementary Figure S1). The migration index was also decreased in PM-pretreated neutrophils from both vitamin D-sufficient and -insufficient participants (Fig. 4d).

### 1α,25-Dihydroxyvitamin D3 enhances the bacterial killing activity of primary human neutrophils isolated from vitamin D-insufficient participants following PM-pretreatment

**Figure 5:**
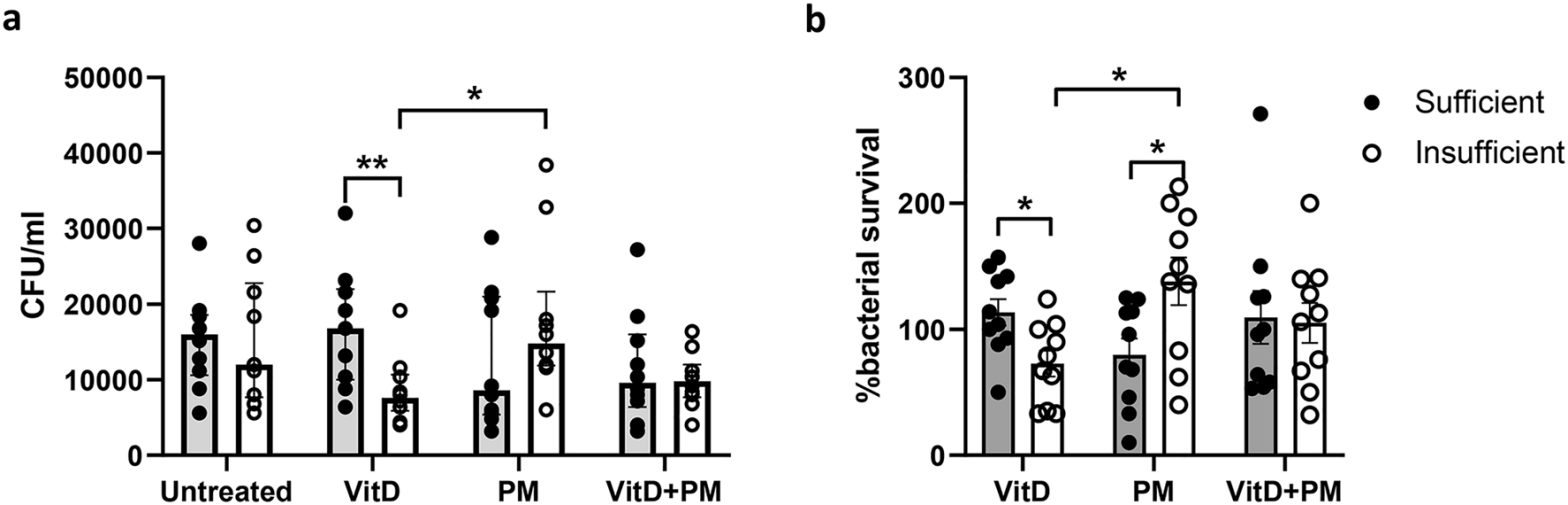
1α,25-Dihydroxyvitamin D3 enhances *E. coli* killing of human neutrophils isolated from vitamin D insufficient participants. Neutrophils isolated from vitamin D-sufficient (n = 10) and vitamin D-insufficient participants (n = 10) were pretreated with 40 μg/ml standard urban PM with or without 100 nM 1alpha,25-dihydroxyvitamin D3 before coculture with live *E. coli* at an MOI of 0.1:1. The cells were lysed for bacterial counting at 3 h. Total bacteria were quantified by standard colony plating, and the results are expressed as percentages of bacterial survival. Each bar indicates the median with the interquartile range of number of bacteria (panel **a**) or % bacterial survival (panel **b**), and each dot represents the value of each participant. Grey and white bars indicate vitamin D-sufficient and vitamin D-insufficient participants, respectively. Statistical analysis was performed using two-way ANOVA with Šídák’s multiple comparisons test. *P < 0.05, No asterisk-nonsignificant.

To investigate the effect of 1α,25-dihydroxyvitamin D3 on the regulation of bacterial killing by PM-pretreated neutrophils, isolated neutrophils from both vitamin D-sufficient and -insufficient participants were pretreated with 1α,25-dihydroxyvitamin D3 alone, PM alone or both 1α,25-dihydroxyvitamin D3 and PM prior to *E. coli* inoculation. No significant difference in bacterial number was found in untreated neutrophils between both participant groups (Fig. 5a). Interestingly, for neutrophils pretreated with vitamin D3 alone, bacterial number and % bacterial survival were significantly reduced when examining cells from vitamin D-insufficient participants (Fig. 5a,b); PM pretreatment alone significantly enhanced % bacterial survival compared with neutrophils from vitamin D-sufficient participants (Fig. 5b). Moreover, when neutrophils were pretreated with both 1α,25-dihydroxyvitamin D3 and PM, % bacterial survival was similar when using cells from both vitamin D-insufficient and vitamin D-sufficient participants (Fig. 5).

## Discussion

Air pollution is the major environmental risk factor for respiratory and cardiovascular disease globally, with the greatest burden experienced in Central and Southeast Asia^31^. Both PM_10_ and PM_2.5_ have been shown to modulate several inflammatory pathways related to the development and exacerbation of chronic cardiopulmonary disease^32,33^, but recent evidence has suggested that they may also be associated with increased infectious disease risk^34,35^. Moreover, a meta-analysis and a population-based time series study have shown a positive association between daily levels of ambient air pollution and hospitalization or mortality of children due to pneumonia^36,37^.

In this study, we found that PM enhanced IL-8 production alone. PM also enhanced IL-8 production in response to LPS stimulation. PM modestly enhanced both CXCR2 expression and the neutrophil migration response at the highest concentration tested. In contrast PM clearly inhibited CXCR2 expression and the migration activity of neutrophils in response to LPS. In particular, preexposure to Chiang Mai PM_2.5_ strongly enhanced IL-8 production in LPS-induced neutrophils compared to LPS alone. However, Chiang Mai PM_2.5_ alone did not induce IL-8 production. Urban PM is total suspended material and contains particles up to 30 µm in diameter. Therefore, the different results might be due to differences in size distribution, composition and extraction methods between the two samples used. Moreover, these data would suggest that the contemporary PM2.5 sample from Chiang Mai is less immunostimulatory than the historic SRM 1648a. Our study suggests that preexposure to PM alters the response of human neutrophils stimulated by LPS, consistent with findings in a recent study using a mouse model^38^.

LPS is a potent microbial inducer of inflammation, and recognition of LPS by the innate immune system is a crucial step in identifying invading gram-negative bacteria and in initiating a protective immune response; any impairment of this process may enhance susceptibility to certain infectious agents. The capacity of PM to increase susceptibility to infections has previously been shown for various gram-positive and gram-negative bacteria such as *Mycobacterium tuberculosis*^39^, *Streptococcus pneumoniae*^40^, and *Pseudomonas aeruginosa*^41^. Furthermore, case-crossover analyses conducted in the United States found that short-term exposure to PM_2.5_ is associated with increased risk of hospital admissions due to septicaemia^42^. However, the current evidence of the effect of PM_2.5_ air pollution on sepsis is insufficient and warrants further investigation. Our results revealed that PM increased IL-8, but reduced IL-8 receptor expression and migration in response to LPS stimulation. This is relevant to a previous study in a mouse model in which PM reduced neutrophil migration to the lungs in response to LPS by decreasing gene expression of TLR-4 and MyD88^38^. Moreover, TLR4-induced loss of IL-8 receptor (CXCR2) was modified by enhanced LPS presentation efficiency^43^. However, there are no direct evidence demonstrated in those studies that LPS is able to bind IL-8 receptor. Together these data suggest that PM interferes with neutrophil migration in response to LPS, an area that warrants more detailed investigation.

Vitamin D3, a lipophilic micronutrient, has been well reported to both attenuate oxidative stress^26,44^ and modulate inflammation^45^, including in neutrophils^46,47^. In CD4 + T cells, it has been shown to increase human α-1-antitrypsin, a serine protease inhibitor that acts to limit excessive lung damage by neutrophil elastase and also has immunoregulatory activities^27,28^. Vitamin D3 also plays a role in the control of airway remodelling, which is a hallmark of severe asthma and COPD^48–50^. Moreover, vitamin D3 has been reported to potentially modify neutrophil function by modulating expression of neutrophil genes via functional vitamin D receptors^51^.

Although the biological roles of vitamin D3 and the adverse effects of PM are well known, the association between vitamin D3 and PM on human neutrophil function has not been addressed. A previous study found that 95 nM of total serum vitamin D was the cut-off for beneficial effects against viral respiratory tract infections^52^, and another in vitro study using human primary neutrophils showed that the effective concentration of vitamin D supplementation for killing activity is 100 nM^24^. The concentrations of 1α,25-dihydroxyvitamin D3 studied here were varied to cover the concentrations from both previous studies and we investigated the potential effects of 1α,25-dihydroxyvitamin D3 on the PM-induced inflammatory response in primary human neutrophils from vitamin D-sufficient individuals as a baseline response. Our data showed that the pretreatment with 100 to 1000 nM of 1α,25-dihydroxyvitamin D3 reduced PM-induced IL-8 production. A high dose at 1000 nM of 1α,25-dihydroxyvitamin D3 likely only occurs during hypercalcemia^53^; however, 100 to 1000 nM of vitamin D is commonly used in in vitro studies. Furthermore, we were able to observe the effect of 1α,25-dihydroxyvitamin D3 from 100 nM.

Overall, our data that PM reduces LPS-induced migration are robust, and this is presumed to be detrimental for the host. Vitamin D is seen as a homeostatic regulator^23^ and the capacity of vitamin D to reduce, but not completely eliminate LPS-induced migration is arguably a good thing to ensure that an inflammatory response is mounted to a pathogen, but is not over-exuberant leading to tissue pathology. In contrast, where PM reduced the LPS-induced migratory response, and then vitamin D reduced this even further we postulate this may compromise the host, leaving it without an appropriate and protective inflammatory response. So, we suggest that a detrimental effect on LPS-induced migration is seen when both PM and vitamin D are present, but not when only vitamin D was present. Future work could test this in an in vivo (animal) challenge model.

Due to sufficient sunlight year-round, the Thai population should not be at risk for vitamin D deficiency, but high levels of insufficiency due to lifestyle and dietary factors have been reported^54^. Our current study is the first to examine the effect of vitamin D on PM-driven human neutrophil function in vitamin D-insufficient participants living in high PM-exposure areas. Interestingly, we found that PM-pretreated neutrophils from vitamin D-insufficient participants had impaired IL-8 production and bacterial killing activity compared with those from vitamin D-sufficient participants. A previous study in mice showed that vitamin D deficiency reduces the immune response, phagocytosis rate, and intracellular killing rate of microglial cells against *E. coli* infection^55^, and severe vitamin D deficiency failed to significantly minimize cellular inflammation in bronchoalveolar lavage samples by instillation of LPS^56^. Thus, the mechanisms underlying this phenomenon need further investigation.

Our major findings on 1α,25-dihydroxyvitamin D3 supplementation in vitro suggest that 1α,25-dihydroxyvitamin D3 improved the bacterial killing activity of PM-pretreated neutrophils from vitamin D-insufficient participants. These data are consistent with another in vitro study of primary human neutrophils demonstrating that vitamin D supplementation enhances *S. pneumoniae* killing^24^. Notably, local nebulization of vitamin D in severely vitamin D-deficient mice failed to attenuate the cytokine storm against LPS challenge and was only effective in mice with normal levels of vitamin D^56^. Moreover, we found that PM-pretreated neutrophils from vitamin D-insufficient participants could not respond to 1α,25-dihydroxy vitamin D3 treatment in terms of decreasing IL-8 production. These observations suggest that the route of vitamin D3 supplementation and establishing the effective dose should be considered in further studies addressing the potential of vitamin D to protect against the harmful effects of PM exposure.

Possible mechanisms that may explain how 1α,25-dihydroxyvitamin D3 regulates PM-driven neutrophil inflammation include the following: (1) inhibition of TLR2, 4, and 9 expression, as previously reported for monocytes^57^; (2) reduction in oxidative stress-mediated inflammation induced via the p38/NF-κB/NLRP3 signalling pathway, as previously reported for human bronchial epithelial cells^25^; and (3) regulation of the cathelicidin antimicrobial peptide gene, as reported for macrophages^58^. Moreover, vitamin D is known to promote expression of the antimicrobial peptides cathelicidin, the alpha-defensins (HNP1–3) and beta-defensins, all mediators of the antibacterial activity from primary human neutrophils for pneumococcal killing^24^. In this study, 100 nM 1α,25-dihydroxyvitamin D3 demonstrated a trend to enhance the killing activity of neutrophils from vitamin D-insufficient individuals (*p* = 0.1823).

Taken together, in vitro short-term exposure to PM, including PM_2.5_ collected in Chiang Mai, Thailand, enhanced IL-8 production stimulated by LPS. In addition, standard urban PM was able to modify the neutrophil inflammatory response to LPS and reduce bacterial killing activity. Importantly, vitamin D3 attenuated IL-8 production and restored bacterial killing in neutrophils from vitamin D-insufficient participants. These results suggest that 1α,25-dihydroxyvitamin D3 is a promising therapeutic target to regulate the PM-driven human neutrophil inflammatory response, and may represent a low cost strategy to protect the population from infectious disease risks in high PM exposure areas.

## Methods

### Participants

We enrolled 20 healthy participants from the Khon Kaen and Chiang Mai provinces. All participants were age matched, and none had signs of acute infectious disease within a 3-months period prior to blood donation. Each participant provided 20 ml of venous blood, which was heparinized, for neutrophil isolation and measurement of free 25-hydroxy vitamin D by ELISA (DIAsource) according to the manufacturer’s instructions.

### Ethics statement

The protocol for blood collection and sample analysis was approved by the Ethics Committee for Human Research of the Faculty of Associated Medical Sciences, Chiang Mai University (AMSEC63EX036) and the Khon Kaen University Ethics Committee for Human Research (HE631315). The study was performed under the approved guidelines and regulations. All subjects provided written informed consent.

### Chiang Mai PM_2.5_ collection and standard reference PM (SRM1648a) sample studied

Two sets of the Tisch model TE-WILBUR-2.5 Low Volume Federal Reference Method (FRM) Ambient Air Particulate Samplers (Tisch Environmental) with the adjusted flow rate at 16.7 l/min were operated to collect PM_2.5_ samples during March 20–27, 2020, at the open rooftop on the 12th floor of Building 2, Faculty of Associated Medical Sciences, Chiang Mai University located in Su Thep Sub district, Mueang Chiang Mai District, Chiang Mai, Thailand (UTM (WGS84) 47Q 497444 E, 2077573 N). This building is approximately 90 m away from Suthep main road, and the height from the rooftop floor to the centre of the inlets is 1.96 m. The activities considered as significant sources of PM during the sampling period were not only from regular traffic and transboundary PM from biomass burning but also PM from the forest fire within the Doi Suthep Mountain region (starting on March 24th), which was 9.31 km from the sampling point. Masses of PM_2.5_ samples on the 47 mm diameter PTFE filters were determined gravimetrically using a 7-digit place ultramicrobalance (Mettler Toledo: METLER UMX 2), and the concentrations were calculated prior to extraction. Extraction was performed by the addition of HPLC grade methanol (3 mL) to the filter in 50 mL falcon tubes, followed by vortexing for 1 min and sonication for 10 min before decanting into fresh falcon tubes. This step was repeated three times with fresh methanol and regular changing of ultrasonic bath water to ensure constant temperature. Filters and extracted PM-methanol solutions were then dried under nitrogen. Extracted filters were then reweighed as described above. The extracted PM_2.5_ was then resuspended as a stock at 1 mg/ml in Milli-Q system (Merck Millipore)-purified water with a resistivity ≥ 18.2 MΩ cm and pretreated with Chelex-100 resin to reduce background metal contamination, as previously described^59^. Stock PM suspensions were stored at − 80 °C prior to use.

Urban particulate matter samples SRM 1648a were purchased from National Institute of Standards and Technology in the USA. The samples were composed of particulate matter collected over a period of 1 year (1976–1977) in the St. Louis (MO, USA) area into a specially designed dust collector. SRM 1648a is a conglomeration of fine and ultrafine particles with the mean particle diameter 5.85 µm^49^.

### Primary human neutrophil isolation and stimulation

Primary human neutrophils were isolated from heparinized venous blood by dextran sedimentation and Ficoll-Paque centrifugation, as previously described^60^. Cell viability was > 98%, as determined by trypan blue exclusion. Unless stated otherwise, isolated neutrophils at 2.5 × 10^6^ cells/ml in RPMI 1640 culture medium (Gibco) were pretreated at 37 °C and 5% CO_2_ for 30 min with Chiang Mai PM_2.5_ or standard (STD) urban PM (SRM Number: 1648a, National Institute of Standards & Technology) at concentrations ranging from 5 to 40 µg/ml. PM-pretreated neutrophils were treated with 100 ng/ml LPS (from *E. coli*, Sigma) for varying periods depending on the indicated measurement. In certain experiments, varying concentrations of 1α,25-dihydroxy vitamin D3 (Sigma‒Aldrich) were added to isolated neutrophils for 30 min during PM pretreatment.

### Measurement of interleukin-8 production

The supernatant from PM-pretreated neutrophil cultures (with or without 10–1000 nM 1α,25-dihydroxy vitamin D3) was harvested 18 h after the addition of LPS and stored at − 80 °C prior to measurement. The IL-8 concentration was assessed in duplicate by ELISA (BD Biosciences) according to the manufacturer’s instructions. The lower limit of detection achieved for this assay is approximately 5 pg/ml.

### Measurement of CXCR2 expression

Following incubations, PM-treated neutrophils, with or without 1α, 25-dihydroxy vitamin D3, were stimulated with 100 ng/ml LPS at 37 °C for 6 h. The suspended cells were then centrifuged and washed with 1 ml 10% FBS in PBS. The pelleted cells were surface stained with anti-human CXCR2-PE (BioLegend) for 30 min at 4 °C. After washing with 10% FBS in PBS, the cells were fixed with 1% paraformaldehyde (Sigma) for 10 min at 4 °C and kept at 4 °C until analysis. Data were acquired by flow cytometry (FACSCalibur; BD Biosciences).

### Measurement of neutrophil migration

PM-pretreated neutrophils at a concentration of 2.5 × 10^6^ cells/ml were incubated in the upper chamber of 3-μm-pore size Transwell plates (Corning Life Sciences), with 100 ng/ml LPS placed in the lower 0.5 ml chamber; plates were incubated at 37 °C for 1 h. Neutrophils transmigrating into the lower chamber with reference beads were counted by flow cytometry (FACSCalibur; BD Biosciences) for 10,000 events gated on the neutrophil population. The absolute count of transmigrated neutrophils was calculated by relating the number of counted cells to the total number of bead events. In certain experiments, isolated neutrophils were pretreated with 40 μg/ml of STD urban PM with or without 100 and 1000 nM of 1α,25-dihydroxyvitamin D3 at 37 °C for 30 min prior to the test for migration. The migration index was calculated by subtracting the absolute count of transmigrated neutrophils to LPS from the medium control and dividing by transmigrated neutrophils to the medium control with the following formula^61^:

$${\text{Migration}}\,\,{\text{index}} = \frac{{{\text{transmigrated}}\,\,{\text{neutrophils}}\,\,{\text{to}}\,\,{\text{LPS}} - {\text{transmigrated}}\,\,{\text{neutrophils}}\,\,{\text{to}}\,\,{\text{control}})}}{{{\text{transmigrated}}\,\,{\text{neutrophils}}\,\,{\text{to}}\,\,{\text{control}}}}$$

### Microorganisms

Pathogenic *E. coli* isolated from clinical samples was grown to mid-logarithmic phase at 37 °C in Luria–Bertani (LB) broth. Bacterial growth was assessed by measuring the optical density at 600 nm. In general, an absorbance index of 1 is equivalent to 10^9^ CFU/ml of bacteria, and the number of viable bacteria (colony-forming units) in inocula was determined by retrospective plating of serial tenfold dilutions on LB agar.

### Total killing assay

Isolated neutrophils were pretreated with 40 μg/ml STD urban PM with or without 100 nM 1α,25-dihydroxyvitamin D3 at 37 °C for 30 min before coculturing with live *E. coli* at an MOI of 0.1:1. The cells were lysed for bacterial counting (initial inoculum time) after 3 h. Total bacteria were quantified by colony plating at the indicated time points, and the results are expressed as percentages of bacterial survival, as calculated by dividing the decreased number of bacteria from PM-treated neutrophils with or without 1α,25-dihydroxyvitamin D3 by the number of bacteria following treatment with non-PM-treated neutrophils.

### Statistics

Comparisons between treatment groups were performed using Friedman’s test with Dunn’s post hoc analysis or two-way ANOVA with Šídák's multiple comparisons test. All analyses were performed using GraphPad PRISM statistical software version 9 (GraphPad 9). *P* values ≤ 0.05 were considered significant.

### Supplementary Information

Supplementary Information.

## Data Availability

The datasets generated and analysed during the current study are available from the corresponding author on reasonable request.
