# Supplementary material for: Vitamin D3 regulates PM-driven primary human neutrophil inflammatory responses
Source: Sci Rep. 2023 Sep 22;13:15850. doi: 10.1038/s41598-023-43252-1 (PMC10516903; doi:10.1038/s41598-023-43252-1)
Supplement: Supplementary file 1 — Supplementary Information. [file 41598_2023_43252_MOESM1_ESM.pdf]

Supplementary information

Vitamin D3 regulates PM-driven primary human neutrophil inflammatory response.

Chidchamai Kewcharoenwong, Aranya Khongmee, Arnone Nithichanon, Tanapat Palaga, Tassanee Prueksasit, Ian Mudway, Catherine Hawrylowicz, Ganjana Lertmemongkolchai*

Supplementary Table S1: Percentage of cell viability of purified neutrophils treated with PM.

PM (µg/ml)	Cell viability by trypan blue		
	30 min	60 min	90 min
0	100	99	100
1.25	100	100	99
2.5	99	98	100
5	100	99	100
10	98	100	99
20	99	100	100
40	99	99	100

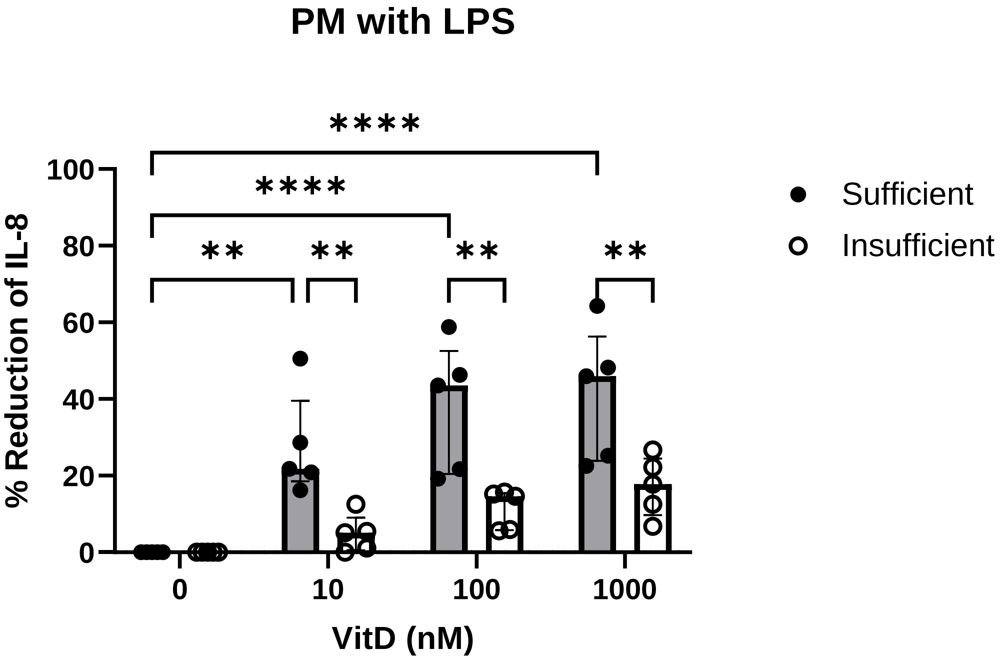

Supplementary Figure S1: Percentage of IL-8 Reduction compared between vitamin D sufficient and insufficient groups. Isolated neutrophils at 2.5 x 106 cells/ml from vitamin D sufficient (n=5) and insufficient participants (n=5) were pretreated with 40 µg/ml of standard urban PM without or with 1α,25-Dihydroxyvitamin D3 (10, 100 and 1000 nM) for 30 min and then stimulated with 100 ng/ml of E. coli LPS. The supernatant of pretreated neutrophil cultures was harvested at 18 h for IL-8 detection. Each bar indicates median with range of % reduction of IL-8 compared with without 1α,25-Dihydroxyvitamin D3 condition (0) and each dot represents the value of each participant. Statistical analysis was performed using Two-Way ANOVA with Šídák's multiple comparisons test among 1α,25-Dihydroxyvitamin D3 pretreatments and between sufficient and insufficient participants. ***P < 0.001, **P < 0.01, No asterisk, non-significant.
